# BMSC-Derived Exosomes Ameliorate Osteoarthritis by Inhibiting Pyroptosis of Cartilage via Delivering miR-326 Targeting HDAC3 and STAT1//NF-*κ*B p65 to Chondrocytes

**DOI:** 10.1155/2021/9972805

**Published:** 2021-11-02

**Authors:** Honggang Xu, Bin Xu

**Affiliations:** Department of Orthopedics, The First Affiliated Hospital of Anhui Medical University, No. 218 Jixi Road, Hefei City, Anhui Province 230022, China

## Abstract

**Background:**

In the past decade, mesenchymal stem cells (MSCs) have been widely used for the treatment of osteoarthritis (OA), and noncoding RNAs in exosomes may play a major role.

**Aim:**

The present study is aimed at exploring the effect and mechanism of miR-326 in exosomes secreted by bone marrow mesenchymal stem cells (BMSCs) on pyroptosis of cartilage and OA improvement.

**Methods:**

Exosomes from BMSCs (BMSC-Exos) were isolated and identified to incubate with OA chondrocytes. Proliferation, migration, specific gene and miR-326 expression, and pyroptosis of chondrocytes were detected. BMSCs or chondrocytes were transfected with miR-326 mimics or inhibitors to investigate the effect of miR-326 in BMSC-Exos on pyroptosis of chondrocytes and the potential mechanism. Finally, a rat OA model was established to verify the effect and mechanism of miR-326 in BMSC-Exos on cartilage of pyroptosis.

**Results:**

Incubation with BMSC-Exos could significantly improve the survival rate, migration ability, and chondrocyte-specific genes (COL2A1, SOX9, Agg, and Prg4) and miR-326 expression of OA chondrocytes and significantly inhibit pyroptosis of chondrocytes by downregulation of the levels of inflammatory cytokines, Caspase-1 activity, and pyroptosis-related proteins such as GSDMD, NLRP3, ASC, IL-1*β*, and IL-18 (*P* < 0.01). PKH26 labeling confirmed the uptake of BMSC-Exos by chondrocytes. Incubation with exosomes extracted from BMSCs overexpressing miR-326 can significantly repress the pyroptosis of chondrocytes, while knockdown of miR-326 had the opposite effect (*P* < 0.01). The same result was also demonstrated by direct interference with the expression level of miR-326 in chondrocytes (*P* < 0.01). In addition, we found that the overexpression of miR-326 significantly inhibited the expression of HDAC3 and NF-*κ*B p65 and significantly promoted the expression of STAT1, acetylated STAT1, and acetylated NF-*κ*B p65 in chondrocytes (*P* < 0.01). The targeted relationship between miR-326 and HDAC3 was verified by dual-luciferase reporter assay. Animal experiments confirmed the mechanism by which miR-326 delivered by BMSC-Exos inhibits pyroptosis of cartilage by targeting HDAC3 and STAT1/NF-*κ*B p65 signaling pathway.

**Conclusion:**

BMSC-Exos can deliver miR-326 to chondrocytes and cartilage and improve OA by targeting HDAC3 and STAT1//NF-*κ*B p65 to inhibit pyroptosis of chondrocytes and cartilage. Our findings provide a new mechanism for BMSC-Exos to treat OA.

## 1. Introduction

Osteoarthritis (OA) is the most common joint disease and the leading cause of degeneration, destruction, and osteogenesis of articular cartilage [1, 2]. Increased prevalence of OA brings an enormous societal burden along with the aging population [3]. The pathogenesis of OA is complex and has a heritable tendency [4]. Due to the interaction of mechanical and biological factors, the imbalance of articular chondrocytes and extracellular matrix (ECM) are the main causes of OA [5]. At present, clinical nondrug therapy, drug therapy, and surgical treatment cannot fundamentally delay the progressive degeneration of articular cartilage during OA. In view of this, a new mesenchymal stem cell- (MSC-) based OA therapy has attracted increasing attention. MSCs can repair cartilage tissue and inhibit the secretion of inflammatory cytokines by chondrocytes and can be directed to differentiate into chondrocytes in vivo. A series of studies confirmed that MSCs can effectively treat OA from different aspects [6–8]. Clinical application has preliminarily proven that MSCs may be the best method to treat traumatic bone and cartilage defects [9]. However, the risk of tumor formation, ethical issues, and transplant rejection remain obstacles to the further clinical application of stem cells [10].

Exosomes are tiny vesicles released into the ECM upon the fusion of multivesicular bodies with the cytoplasmic side of the plasma membrane, which are shaped like discs and have a diameter of 30-150 nm [11, 12]. Exosomes contain a variety of proteins, lipids, and nucleic acids with important functions that can be transferred to other cells to affect the function of the recipient cells. A growing body of data suggests that MSCs can inhibit the development of disease by secreting exosomes carrying proteins, miRNAs, long noncoding RNAs, or other small molecules [13]. Studies have found that exosomes derived from MSCs can promote chondrocyte proliferation, inhibit apoptosis, enhance chondrogenesis, inhibit cartilage degradation, promote cartilage repair, and relieve osteoarthritis by balancing the synthesis and degradation of chondrocyte ECM [14–17]. MSC-derived exosomes are also widely recognized for their role in protecting cartilage degeneration and promoting cartilage repair and regeneration and the treatment of osteoarthritis [18–21]. A recent study showed that bone marrow mesenchymal stem cell- (BMSC-) derived exosomes protected cartilage damage and alleviated knee pain in osteoarthritis model rats [22]. In addition, BMSC-derived exosomes from congenital polydactyly tissue alleviate osteoarthritis by promoting chondrocyte proliferation [23]. However, the specific mechanism of BMSC-derived exosomes against osteoarthritis remains unclear.

Pyroptosis, including caspase-dependent programmed cell death and proinflammatory changes, is a combined process of apoptosis and necrosis [24, 25]. Unlike other forms of programmed cell death, pyroptosis is closely associated with inflammation. Pyrolysis is associated with risk factors for OA and is involved in cartilage degeneration, synovial changes, and OA-induced pain and plays an important role in OA [26]. There are also evidences that inhibition of pyroptosis of chondrocytes can ameliorate cartilage degeneration and arthritis [27, 28]. MSCs have been shown to interfere with pyroptosis during cerebral ischemia/reperfusion [29] and acute liver failure [30]. Moreover, it has been reported that exosomes of MSCs can protect cardiomyocytes from hypoxia/reoxygenation-induced pyroptosis [31]. However, in OA-related studies, there has been no report on the effect of MSC- or BMSC-derived exosomes on pyroptosis of chondrocytes.

Exosomes secreted by a variety of different cells have therapeutic or mitigating effects through miR-326 in inflammatory bowel disease [32], relapsing-remitting multiple sclerosis [33], and hepatocellular carcinoma [34] and are associated with inflammation and NF-*κ*B pathway. MiR-326 has also been proved to be involved in the regulation of pyroptosis in Parkinson's disease [35]. In this study, we will explore the effect of miR-326 in BMSC-derived exosomes (BMSC-Exos) on pyroptosis of chondrocytes and the potential mechanism and discuss the possibility of BMSC-Exos in the treatment of OA.

## 2. Materials and Methods

### 2.1. Cell Culture and Treatment

Bone marrow was taken from the rat femur and washed with *α*-minimum essential medium (*α*-MEM, Gibco, USA) to isolate bone marrow mesenchymal stem cells (BMSCs). BMSCs cultured with *α*-MEM contained 10% fetal bovine serum, 100 U/mL penicillin (Solarbio, China), and 10 mg/mL streptomycin (Solarbio) at 37°C with 5% CO_2_. Cartilage tissue was collected from the knee joints of rats, washed with phosphate-buffered saline (PBS), cut into pieces, and digested with collagenase II (col II) to obtain chondrocytes. Isolated mouse chondrocytes and BMSCs were cultured in Dulbecco's Modified Eagle Medium (DMEM) supplemented with 10% fetal bovine serum (Gibco, USA) and 100 U/mL penicillin at 37°C with 5% CO_2_. Subculture was carried out when the cell confluence rate was close to 80%. Chondrocytes was stimulated by 10 ng/mL interleukin-1*β* (IL-1*β*) for 24 h to construct the osteoarthritis model as previously described [22].

### 2.2. Exosomes Collection and Detection

BMSCs were cultured in exosome-free media in the initial. Media from cells and PBS-washed cells was collected and together centrifuged for 5 minutes, and then, the supernatant was centrifuged for 20 minutes. Exosomes were isolated with the ExoQuick-TC™ system (System Bioscience, USA) according to the protocol. Exosomal protein was measured via a BCA assay, and 10 *μ*g exosomes were placed on a copper grid. Exosomes were skillfully separated to form a thin layer before a thin layer of 2% uranyl acetate is added. Grids were allowed to dry overnight, and transmission electron microscopy (TEM) was performed the next day. The size distribution of the exosomes was measured using Nanosizer™ technology (Malvern Instruments, UK), and was analysed using Zetasizer software (Malvern). After OA modeling, chondrocytes were treated with 10 *μ*g/mL BMSC-Exos for 12 h at 37°C [22].

### 2.3. Exosome Labeling

Exosome suspension (100 *μ*L) was mixed with 1 mL of dilution C-diluted PKH67 (Sigma, USA) for incubation at room temperature for 4 min. The staining was terminated by adding 1 mL of 0.5% Bovine Serum Albumin (BSA, Gibco), and the exosomes were reextracted. The observation under a fluorescence microscope showed that the exosomes were stained by PKH67 (red). Chondrocytes were stained with DAPI (Sigma) in blue. PKH67-labeled exosomes were incubated with chondrocytes for 12 h before locating the exosomes.

### 2.4. Cell Transfection

BMSCs were transfected with miR-326 mimics (5′-CCCCCGTCCCGGAAACACTT-3′) for overexpression the miR-326 level, miRNA mimics control (mimics NC), miR-326 inhibitor (5′-TTACAAAGGCCCTGCCCTGCCCCC-3′) for knockdown the miR-326 level, and miRNA inhibitor control (inhibitor NC) (GenePharma, China). HDAC3 overexpression plasmid pcDNA3.1-HDAC3 (HDAC3 OE) and its negative control plasmid (NC OE) purchased from GenePharma were used to transfect chondrocytes. Cell transfections were performed using the transfection reagent Lipofectamine 2000 (Invitrogen, USA) according to manufacturer's instructions. In brief, cells (3 × 10^5^) were seeded into 6-well plates for cell transfection upon cell fusion of 60~80%. 500 *μ*L Opti-MEM culture medium (Gibco, USA) containing 5 *μ*L Lipofectamine 2000 and 5 *μ*L miR-326 mimics, inhibitors, or controls was added to each well and cultured in an incubator at 37°C for 6 h. The captured cells were treated with 100 nM Trichostatin A or 50 *μ*mol/L AG490 at room temperature for 4 h.

### 2.5. Western Blot Assay

Chondrocytes or cartilages were collected and disrupted with RIPA cleavage buffer (Thermo Fisher Scientific). Proteins were extracted from exosomes using a Total Exosome Protein Isolation Kit (Invitrogen, Carlsbad, CA, USA) following the instructions supplied. The lysates were collected after centrifugation, and the protein concentration was quantified with BCA kit (Thermo Fisher Scientific). 50 mg of protein was added to sodium dodecyl sulfate polyacrylamide gel electrophoresis (SDS-PAGE) and then were transferred onto polyvinylidene difluoride (PVDF) membranes (Millipore, USA). These membranes were blocked in 5% nonfat milk for 1 hour at room temperature and then were treated as the antibody protocol described overnight at 4°C. The following antibodies purchased from Abcam and Cell Signal Technology (USA) were used: anti-CD9 (ab92726, Abcam), anti-CD63 (ab217345, Abcam), anti-CD81 (ab109201, Abcam), anti-HDAC3 (ab32369, Abcam), anti-STAT1 (ab230428, Abcam), anti-acetyl-STAT1 (361250, USBiological, USA), anti-NF-*κ*B p65 (8242, Cell Signaling), anti-acetyl-NF-*κ*B p65 (12629, Cell Signaling), anti-NLRP3 (13158S, Cell Signaling), anti-ASC (67824T, Cell Signaling), anti-GSDMD (ab209845, Abcam), anti-Caspase-1 (Nbp2-15713, Novus Biologicals, USA), anti-IL-1*β* (12242, Cell Signaling), anti-IL-18 (57058, Cell Signaling), and anti-GAPDH (ab8245, Abcam). Moreover, the respective secondary antibody was used to incubate these membranes according to protocol. The protein bands were quantified with ECL system (Thermo Fisher Scientific) and were analyzed by Image J software.

### 2.6. Quantitative Reverse Transcription-Polymerase Chain Reaction (qRT-PCR)

The total RNA of cells or tissue was extracted with Trizol (Takara, Japan). RNA reverse transcription kit (Takara) was used to reverse transcribe cDNA, and 1 *μ*g cDNA and SYBR Green RT-PCR kit (Takara) were taken for qRT-PCR. The reaction conditions were predenaturation at 95°C for 3 min, 40 cycles (95°C 30 s, 58°C 45 s) and extended at 72°C for 6 min. Primers synthesized by Shanghai Sangon Biotech Co., Ltd. (China) were used. GAPDH and U6 were used as internal references for mRNA and miRNA, respectively. The 2^−ΔΔCt^ method was used to calculate the relative expression of targets. Repeat at least 3 times for each sample.

### 2.7. CCK-8 Assay

The CCK-8 kit was purchased from Boster Biological Technology Co., Ltd. (China) and operated according to supplier's instructions. Cells in the logarithmic growth phase were seeded into 96-well plates. After 1, 3, 5, and 7 days of culture, the optical concentration (OD) values at 450 nm were measured with a microplate analyzer (Shimadzu, Japan).

### 2.8. Wound Healing Assay

Before the experiment, the medium was changed to serum-free medium. Pipette tip was used to create cell scratches, and the cell surface was washed once with serum-free medium. The cells were observed under a microscope (Olympus, Japan) and photographed, and the positions of cells in the photos were recorded, denoted as 0 h. Instead, serum-free culture was conducted in an incubator at 37°C and 5% CO_2_ for 24 h. Take pictures and calculate the wound closure of cells using Image J software.

### 2.9. Immunofluorescence Assay

The articular cartilage was embedded in paraffin and sliced into 5 *μ*m slices. Cells or tissues were fixed with methanol and permeabilized with 0.1% Triton X-100 in PBS for 20 min. The tissues were incubated with GSDMD (ab209845, Abcam) or cleaved Caspase-1 (cl-Caspase-1, af4005, Affinity Biosciences, USA) and Collagen II (Col II, MA5-12789, Invitrogen, USA) primary antibody (Cell Signal Technology), and cells were incubated with GSDMD or cleaved Caspase-1 primary antibody overnight at 4°C and then incubated in fluorochrome-conjugated or normal secondary antibodies for 2 h at room temperature. DAPI was used to counterstain the cell nuclei. Images were captured with a laser confocal microscope (Olympus, Japan) in five different fields for each sample.

### 2.10. Caspase-1 Activity Assay

Caspase-1 activity assay kit was purchased from Beyotime Biotechnology Company (China). The cells or tissues were lysed with lysate, and the lysate was collected and centrifuged for 10 min. After the supernatant was taken to determine the protein concentration, the same amount of samples were placed in a 96-well microtitration plate, treated with 20 ng Ac-DEVD-pNA at 37°C and incubated overnight. The absorbance at 405 nm was measured with a microplate analyzer to obtain the content of pNA generated, and the activity of Caspase-1 contained in the unit weight protein was calculated.

### 2.11. Enzyme-Linked Immunosorbent Assay (ELISA)

Concentrations of IL-1*β*, IL-6, IL-18, and TNF-*α* in cell culture medium or rat articular cartilage were measured with rat ELISA Kits purchased from Sigma-Aldrich (USA) according to supplier's protocols. Absorbance was measured at 450 nm wavelength.

### 2.12. NF-*κ*B p65 DNA-Binding Assay

NF-*κ*B p65 DNA-binding activity in chondrocytes or cartilage nuclear extracts was determined with a transcription factor binding assay colorimetric ELISA kit (Cayman Chemical, USA) according to manufacturer's protocol as previously described [36]. Briefly, NF-*κ*B p65 present in the nuclear extract binds specifically to the NF-*κ*B response element (a double-stranded DNA sequence containing the NF-*κ*B response elements immobilized to the wells of a 96-well plate), and the DNA-protein complex was detected by addition of a specific primary antibody directed against NF-*κ*B p65. A secondary antibody conjugated to HRP was added, and the absorbance was read at 450 nm.

### 2.13. Dual-Luciferase Reporter Assay

Wide sequence (WT-HDAC3) and mutant sequences (MUT-HDAC3) were designed and synthesized by GenePharma. Sequences were inserted into a luciferase reporter vector (pGL3-Basic). Vectors were cotransfected with miR-326 mimic or miRNA mimic NC into HEK293T cells. Cells were lysed with 100 *μ*L of lysis buffer in a shaking bed at room temperature for 20 min. Cell suspension was incubated with luciferase solution (Promega, USA) before the intensity of firefly luciferase was determined. Stop&Glo reagent I from Promega was fully mixed with cell suspension, and then, the intensity of Renilla luciferase was measured. The activity of Renilla luciferase was considered as an internal control. The relative luciferase activity shall be the ratio of firefly luciferase intensity and Renilla luciferase intensity. Triplicate wells were set for each group.

### 2.14. Construction of the Animal OA Model and Grouping

All procedures were performed with the approval of the Animal Ethics Committee of Anhui Medical University, in accordance with the National Institutes of Health Guide for the Care and Use of Laboratory Animals. Twenty-four adult male Sprague Dawley rats (weighing 200~220 g) were anesthetized with 2.5% isoflurane. Eighteen rats were performed the unilateral intra-articular injection with 8% sodium iodoacetate (Sangon Biotech, China) to produce the OA change of the knee, and the rats in the sham group were injection with normal saline. One week later, intra-articular injection of BMSC-Exos (40 *μ*g/100 *μ*L) was performed in the OA rats once a week [22] (*n* = 6, OA+BMSC-Exo group and OA+BMSC-miR-326-Exo group). At 6 weeks after surgery, the knee joint specimens of rats were collected for histologic analysis. Gross scores of cartilage repair were made according to the International Society for Chondroprosthesis (ICRS) criteria.

### 2.15. Histologic Analysis

At 6 weeks after treatment, the rats were sacrificed, and articular cartilage samples were collected. After fixation with paraformaldehyde and decalcified in 10% EDTA, the tissues were embedded in paraffin and sectioned into a 5 *μ*m thick section. Sections were made perpendicular to the defect area. The selected sections were deparaffinized in xylene, rehydrated through a graded series of ethanol washes, and followed by hematoxylin and eosin (H&E) and Safranin O/Fast Green staining (Solarbio). Then, the sections were stained with freshly prepared Weigert solution for 5 min and differentiated with acid differentiation solution for 15 s. The sections were immersed in Fast Green and Safranin O staining solution for 5 min and 2 min, respectively, and washed with distilled water for 1 min. The sections were washed with weak acid solution for 1 min and washed with distilled water for 1 min. The sections were dehydrated and transparent and finally observed under a light microscope.

### 2.16. Statistical Analysis

All data are presented as the means ± standard deviation (SD). The data statistical significance analyses and statistical mapping were performed using Prism GraphPad 8.0 software. Data were analyzed using one-way ANOVA and Student's *t*-test. *P* < 0.05 was considered statistically significant.

## 3. Results

### 3.1. BMSC-Exos Inhibit Pyroptosis and Maintain Chondrocyte Homeostasis of Rat OA Chondrocytes

**Figure 1:**
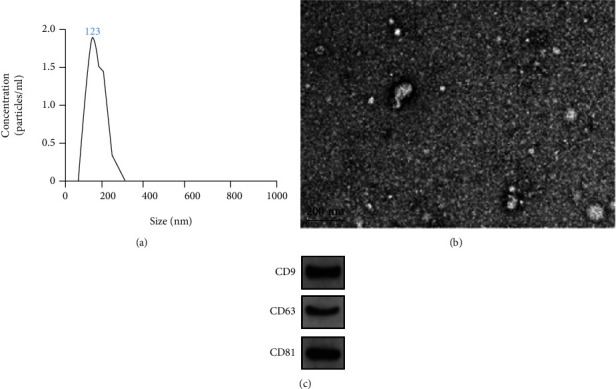
Identification of exosomes by NTA, TEM, and Western blot. (a) NTA was used to determine the diameter distribution of extracted exosomes at about 123 nm; (b) the morphology of exosomes was observed by TEM; (c) exosome marker proteins CD9, CD63, and CD81 were detected by Western blot.

To investigate the effect of BMSC-derived exosomes (BMSCs-Exos) on chondrocytes, exosomes derived from BMSCs were isolated and identified by nanoparticle tracking analysis (NTA), TEM, and Western blot. The diameter distribution of exosomes was analyzed by NTA, and the particles ranged around 123 nm in diameter (Figure 1(a)). Isolated exosomes were examined for size and structure via transmission electron microscopy (Figure 1(b)). Subsequently, the expressions of CD9, CD63, and CD81 exosome markers in the isolated exosomes were detected by Western blot (Figure 1(c)). Based on these, it was concluded that exosomes of BMSCs were successfully isolated and purified.

**Figure 2:**
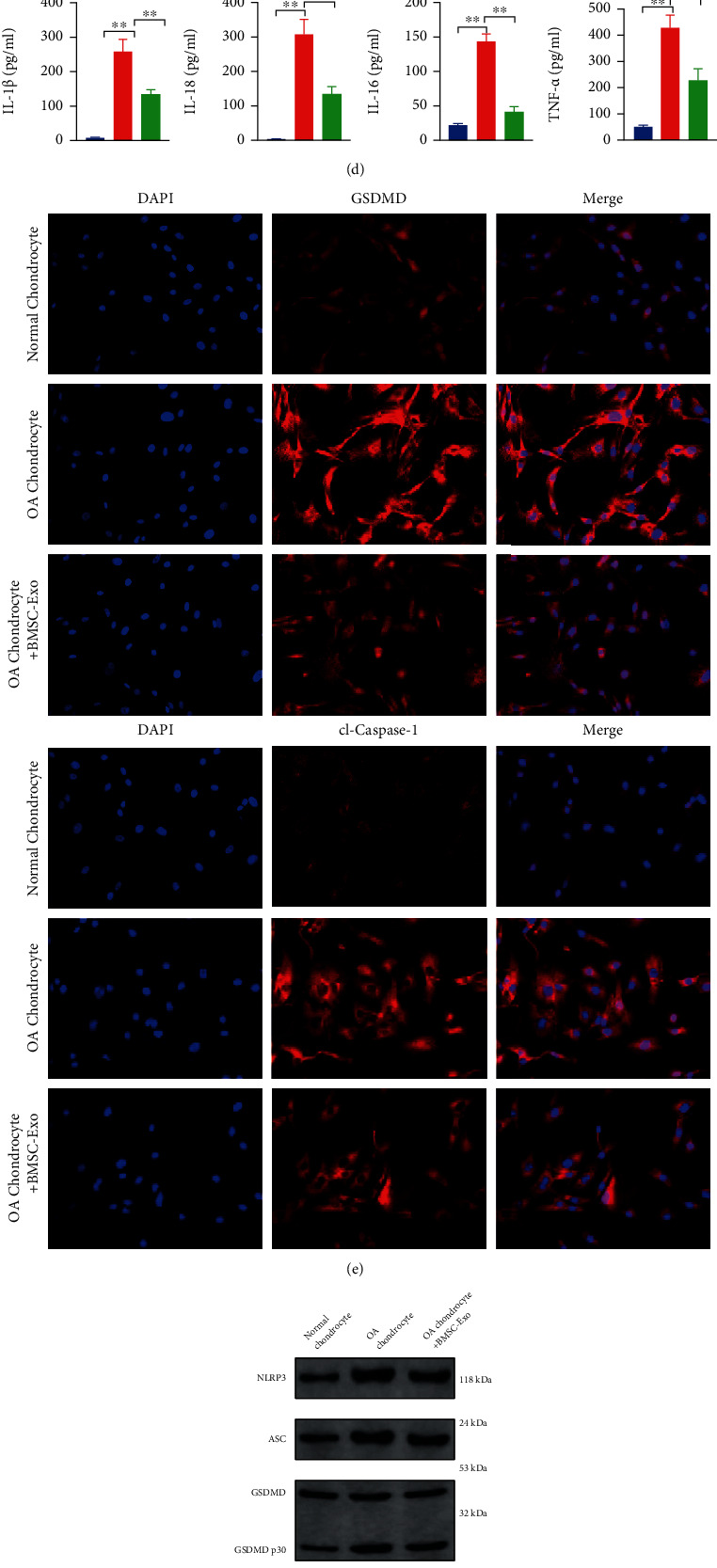
BMSC-Exos inhibit the pyroptosis and maintain chondrocyte homeostasis of OA chondrocytes. (a) Incubation of BMSC-Exos reversed the slow proliferation of chondrocytes induced by OA; (b) incubation of BMSC-Exos reversed the reduction of chondrocyte migration induced by OA; (c) incubation of BMSC-Exos reversed the reduction of chondrogenic characteristic genes (COL2A1, SOX9, Agg, and Prg4) mRNA expression in chondrocytes induced by OA; (d) incubation of BMSC-Exos inhibited the increase of proinflammatory cytokines in chondrocyte culture medium induced by OA; (e) immunofluorescence results showed that BMSC-Exos incubation inhibited the OA-induced increased distribution of GSDMD and cl-Caspase-1 in chondrocytes; (f) BMSC-Exos incubation inhibited the OA-induced increased of Caspase-1 activity in chondrocytes; (g) results of Western blot showed that BMSC-Exos incubation inhibited the OA-induced increased expression of pyroptosis-related proteins (NLRP3, ASC, GSDMD, Caspase-1, IL-1*β*, and IL-18) in chondrocytes; ^∗∗^*P* < 0.01.

After that, isolated BMSC-Exos were used to incubate OA chondrocytes to explore the effect of BMSC-Exos on pyroptosis of chondrocytes. Compared with normal chondrocytes, the survival rate and migration ability of OA chondrocytes decreased significantly, and BMSCs-Exos incubation can reverse the decrease of proliferation and migration ability of OA chondrocytes (*P* < 0.01, Figures 2(a) and 2(b)). The mRNA expression levels of COL2A1, SOX9, Agg, and Prg4 showed that the expression levels of chondrogenic specific genes in OA chondrocytes were significantly decreased compared with normal chondrocytes, and BMSCs-Exos incubation could significantly increase the level of chondrogenic specific genes in OA chondrocytes (*P* < 0.01, Figure 2(c)).

To investigate the effect of BMSCs-Exos on pyroptosis of OA chondrocytes, we detected the expression levels of the key genes of pyroptosis by immunofluorescence, ELISA, and Western blot. The levels of IL-1*β*, IL-18, IL-6, and TNF-*α* in cell culture medium were detected by ELISA, and all proinflammatory cytokines in OA chondrocyte culture medium were increased and significantly decreased after BMSCs-Exos were incubated (*P* < 0.01, Figure 2(d)). Results of immunofluorescence showed that OA significantly increased the levels of GSDMD and cleaved Caspase-1 in chondrocytes, and BMSCs-Exos incubation significantly decreased the levels of them in chondrocytes (*P* < 0.01, Figure 2(e)). The same trend was also shown in Caspase-1 activity assay, that is, OA increased the Caspase-1 activity of chondrocytes, and BMSC-Exos incubation reversed the OA-induced Caspase-1 activation (*P* < 0.01, Figure 2(f)). Western blot detected the expression levels of pyroptosis-related proteins in each group and found that the levels of NLRP3, ASC, GSDMD, Caspase-1, IL-1*β*, and IL-18 were significantly decreased after BMSC-Exos were incubated (*P* < 0.01, Figure 2(g)).

### 3.2. BMSC-Exos Inhibited Pyroptosis by Delivering miR-326 into Chondrocytes

**Figure 3:**
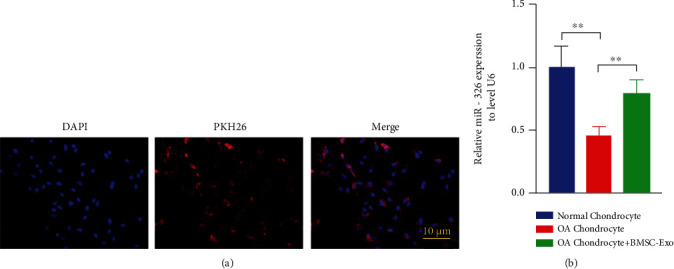
BMSC-Exos delivered miR-326 to OA chondrocytes. (a) PKH26 markers showed BMSC-Exos uptake by chondrocytes; (b) incubation of BMSC-Exos reversed OA-induced reduction of miR-326 levels in chondrocytes; ^∗∗^*P* < 0.01.

BMSC-Exos were labeled with PKH26 and incubated with chondrocytes for 12 h. The nuclei were located by DAPI staining, and fluorescence microscopy showed that exosomes could be ingested by chondrocytes (Figure 3(a)). RT-PCR showed that miR-326 expression level in OA chondrocytes was significantly decreased compared with normal chondrocytes and increased significantly after BMSC-Exos incubation (*P* < 0.01, Figure 3(b)). These results suggest that BMSC-Exos deliver miR-326 into chondrocytes.

**Figure 4:**
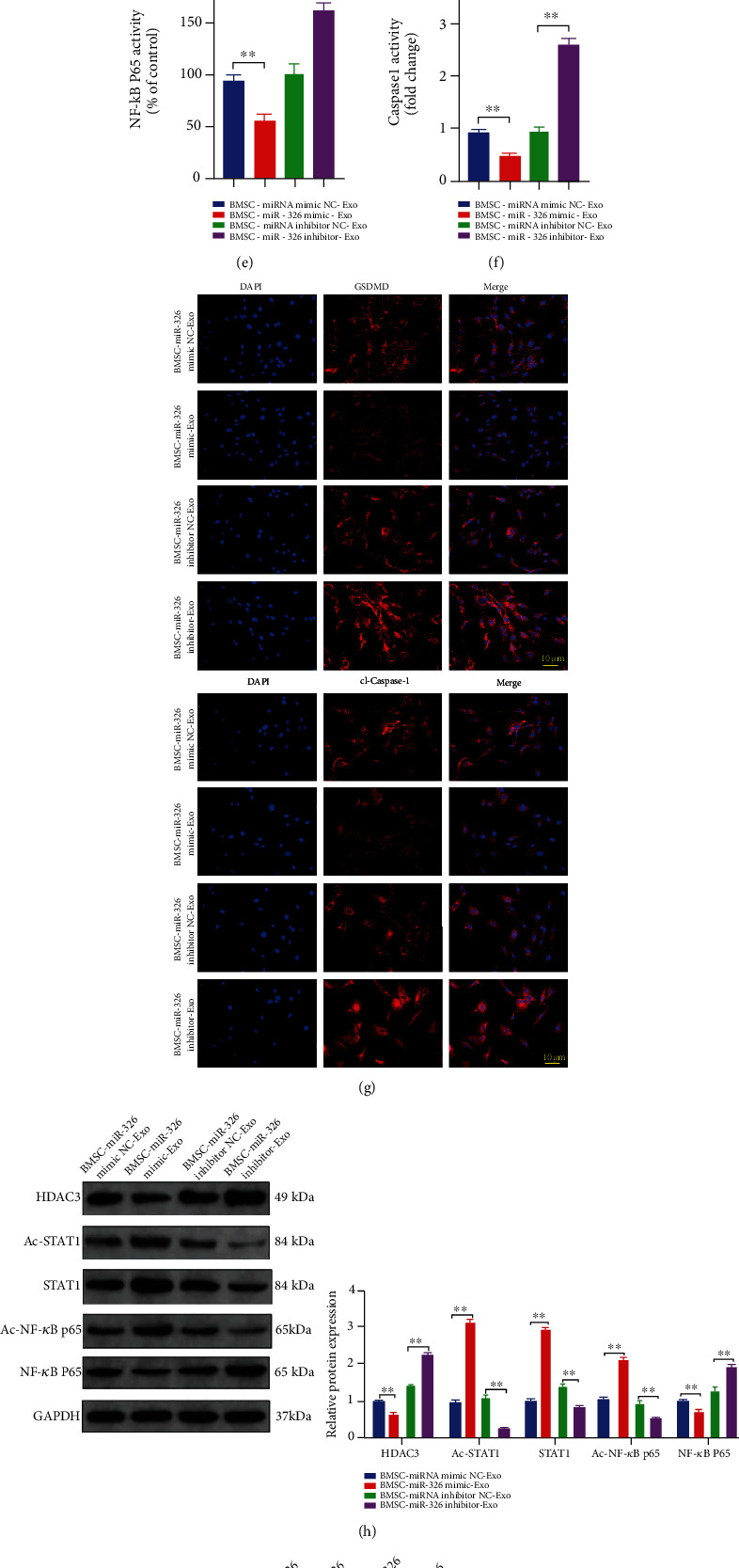
Exosomes extracted from miR-326-overexpressed BMSCs significantly inhibited pyroptosis, HDAC3 expression, and STAT1/NF-*κ*B p65 signaling pathway activation in OA chondrocytes, while silencing miR-326 had the opposite effect. (a) Exosomes extracted from miR-326-overexpressed BMSCs significantly promoted proliferation of OA chondrocytes, while silencing miR-326 had the opposite effect; (b) exosomes extracted from miR-326-overexpressed BMSCs significantly promoted migration of OA chondrocytes, while silencing miR-326 had the opposite effect; (c) exosomes extracted from miR-326-overexpressed BMSCs significantly increased the mRNA levels of chondrogenic specific genes (COL2A1, SOX9, Agg, and Prg4) in OA chondrocytes, while silencing miR-326 had the opposite effect; (d) exosomes extracted from miR-326-overexpressed BMSCs significantly decreased the levels of proinflammatory factors (IL-1*β*, IL-18, IL-6, and TNF-*α*) in OA chondrocytes, while silencing miR-326 had the opposite effect; (e) exosomes extracted from miR-326-overexpressed BMSCs significantly decreased DNA-binding activity of NF-*κ*B p65 in OA chondrocytes, while silencing miR-326 had the opposite effect; (f) exosomes extracted from miR-326-overexpressed BMSCs significantly decreased Caspase-1 activity in OA chondrocytes, while silencing miR-326 had the opposite effect; (g) immunofluorescence results showed that exosomes extracted from miR-326-overexpressed BMSCs significantly decreased the levels of GSDMD and cl-Caspase-1 in OA chondrocytes, while silencing miR-326 had the opposite effect; (h) Western blot results showed that exosomes extracted from miR-326-overexpressed BMSCs significantly decreased the level of HDAC3 and activated the STAT1/NF-*κ*B p65 signaling pathway in OA chondrocytes, while silencing miR-326 had the opposite effect; (i) Western blot results showed that exosomes extracted from miR-326-overexpressed BMSCs significantly decreased the levels of pyroptosis-related proteins (NLRP3, ASC, GSDMD, Caspase-1, IL-1*β*, and IL-18) in OA chondrocytes, while silencing miR-326 had the opposite effect; ^∗^*P* < 0.01, ^∗∗^*P* < 0.01.

To verify the regulatory effect of miR-326 in BMSC-Exos on chondrocyte pyroptosis, miR-326 mimics and inhibitors were constructed and transfected into BMSCs. Exosomes from transfected BMSCs were extracted and used to treat OA chondrocytes. We found that the overexpression of miR-326 exosomes enhanced the proliferation and migration of OA chondrocytes, while the inhibition of miR-326 exosomes weakened (*P* < 0.01, Figures 4(a) and 4(b)). RT-PCR was performed to detect the level of miR-326 in each group of chondrocytes, and it was found that treatment with BMSCs-Exos that promoted or inhibited miR-326 could correlative promote or inhibit the level of miR-326 in chondrocytes (*P* < 0.01, Figure 4(c)), confirming that BMSCs-Exos could deliver miR-326 into chondrocytes. In addition, the mRNA expression level of chondrocyte-specific genes was significantly increased by the overexpression of miR-326 in BMCS-Exos, while the expression levels of chondrocyte-specific genes (COL2A1, SOX9, Agg, and Prg4) were significantly decreased by the inhibition of miR-326 (*P* < 0.01, Figure 4(c)). The detection of inflammatory cytokine levels in the cell culture medium of each group showed that incubation of BMSCs-Exos with overexpression of miR-326 significantly reduced the level of proinflammatory cytokines (IL-1*β*, IL-18, IL-6, and TNF-*α*) in the extracellular fluid of chondrocytes, while silence of miR-326 significantly increased the level of proinflammatory cytokines (*P* < 0.01, Figure 4(d)). The levels of GSDMD and cleaved Caspase-1 were detected by immunofluorescence, and it was found that overexpression of miR-326 significantly reduced the levels of GSDMD and active Caspase-1, while silencing miR-326 was the opposite (*P* < 0.01, Figure 4(g)). Detection of Caspase-1 activity also showed the same results, and Caspase-1 activity in chondrocytes was negatively correlated with miR-326 level in BMSCs, from which exosomes were extracted (*P* < 0.01, Figure 4(f)). The detection of the expression level of pyroptosis-related proteins in cells revealed that the overexpression of miR-326 in BMSCs-Exos significantly reduced the expression level of pyroptosis-related proteins in OA chondrocytes, whereas silencing miR-326 had the opposite effect (*P* < 0.01, Figure 4(i)).

The above results suggest that BMSC-Exos affect chondrocyte homeostasis and pyroptosis through miR-326. We attempted to explore downstream factors that might be targeted by miR-326, and we detected the expression levels of HDAC3 and STAT1/NF-*κ*B p65 in each group. BMSC-Exos overexpressing miR-326 significantly reduced the expression of HDAC3 and NF-*κ*B p65, and DNA-binding activity of NF-*κ*B p65 in chondrocytes, but significantly increased the levels of acetyl-STAT1, STAT1, and acetyl-NF-*κ*B p65 (*P* < 0.01, Figures 4(c), 4(f), and 4(h)). Inhibition of miR-326 had the opposite effect (*P* < 0.01, Figures 4(c), 4(f), and 4(h)), suggesting that miR-326 may affect chondrocyte pyroptosis through HDAC3 and STAT1/NF-*κ*B p65 signaling pathways.

### 3.3. MiR-326 Inhibits Pyroptosis of Chondrocytes by Targeting HDAC3 and Activating the STAT1/NF-*κ*B p65 Signaling Pathway

**Figure 5:**
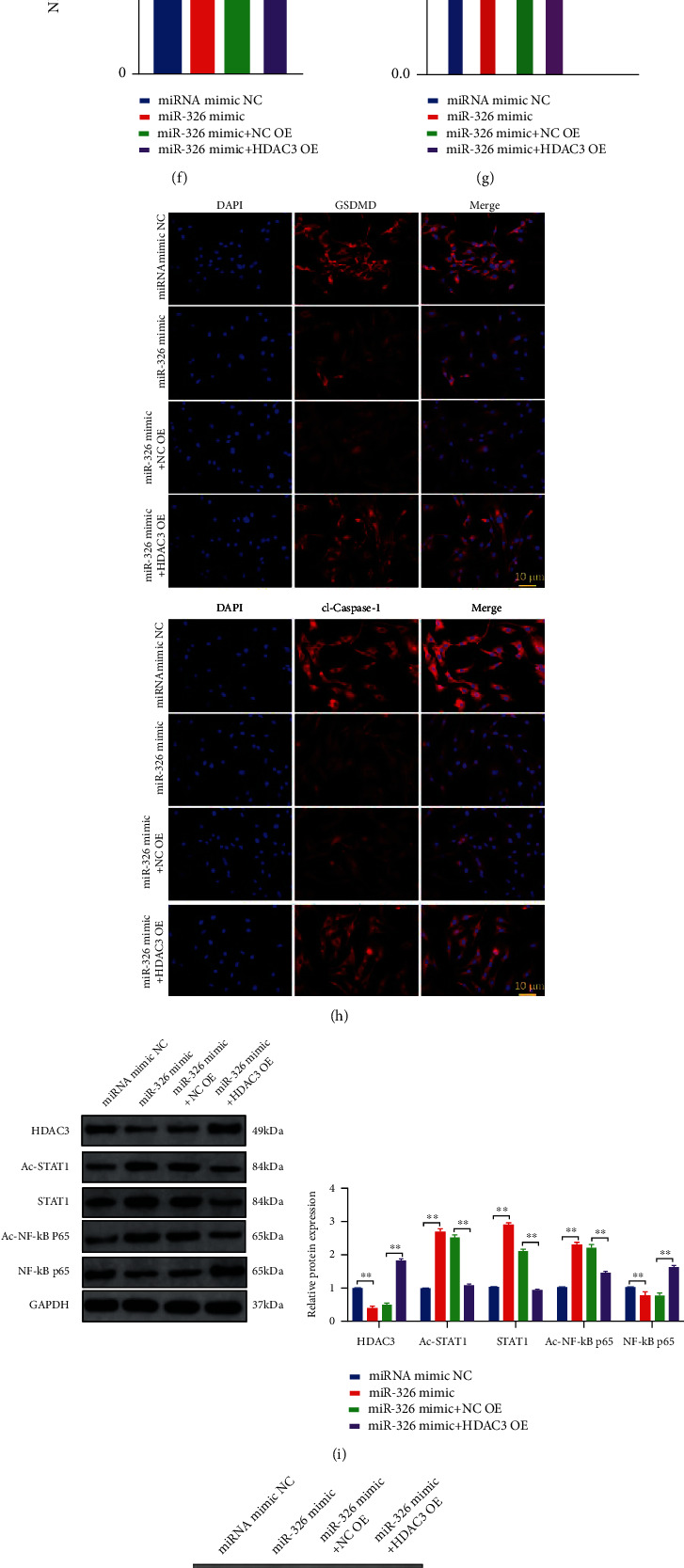
MiR-326 inhibited chondrocyte pyroptosis by targeting HDAC3 and activating the STAT1/NF-*κ*B p65 signaling pathway. (a) Overexpression of HDAC3 could counteract the accelerated proliferation of OA chondrocytes induced by miR-326 mimics; (b) overexpression of HDAC3 could counteract the enhanced migration of OA chondrocytes induced by miR-326 mimics; (c) overexpression of HDAC3 could counteract the increased mRNA levels of chondrogenic genes (COL2A1, SOX9, Agg, and Prg4) induced by miR-326 mimics in OA chondrocytes; (d) the dual luciferase gene report confirmed the targeting relationship between miR-326 and HDAC3; (e) overexpression of HDAC3 could reverse the reduction of proinflammatory factors (IL-1*β*, IL-18, IL-6, and TNF-*α*) in OA chondrocyte culture medium induced by miR-326 mimics; (f) Overexpression of HDAC3 could reverse the decrease of DNA-binding activity of NF-*κ*B p65 in OA chondrocytes induced by miR-326 mimics; (g) overexpression of HDAC3 could reverse the decrease of Caspase-1 activity in OA chondrocytes induced by miR-326 mimics; (h) immunofluorescence results showed that overexpression of HDAC3 could reverse the decrease level of GSDMD and cleaved Caspase-1 in OA chondrocytes induced by miR-326 mimics; (i) Western blot results showed that overexpression of HDAC3 could counteract the activation of STAT1/NF-*κ*B p65 signaling pathway in OA chondrocytes induced by miR-326 mimics; (j) Western blot results showed that overexpression of HDAC3 could reverse the decrease level of pyroptosis-related proteins (NLRP3, ASC, GSDMD, Caspase-1, IL-1*β*, and IL-18) in OA chondrocytes induced by miR-326 mimics; ^∗^*P* < 0.01, ^∗∗^*P* < 0.01.

To directly verify the effect of miR-326 on chondrocytes and explore its mechanism, we transfected OA chondrocytes with miR-326 mimics and HDAC3 overexpression vector (HDAC3 OE). Overexpression of miR-326 could significantly enhance the proliferation and migration ability of OA chondrocytes, while overexpression of HDAC3 could counteract the promoting effect of miR-326 mimics (*P* < 0.01, Figures 5(a) and 5(b)). MiR-326 mimic could significantly increase the expression level of chondrocyte-specific genes, and overexpression of HDAC3 can significantly reduce the mRNA expression levels of COL2A1, SOX9, Agg, and Prg4 increased by miR-326 (*P* < 0.01, Figure 5(c)). In addition, miR-326 mimic could significantly inhibit the release of proinflammatory factors (IL-1*β*, IL-18, IL-6, and TNF-*α*), reduce GSDMD and cleaved Caspase-1 levels of chondrocytes and Caspase-1 activity, and decrease the expression level of pyroptosis-related proteins (NLRP3, ASC, GSDMD, Caspase-1, IL-1*β*, and IL-18) (*P* < 0.01, Figures 5(e), 5(g), 5(h), and 5(j)). Overexpression of HDAC3 could reverse all above indexes and promote chondrocyte pyroptosis (*P* < 0.01, Figures 5(e), 5(g), 5(h), and 5(j)), suggesting that miR-326 inhibits chondrocyte pyroptosis by inhibiting HDAC3 expression. The dual luciferase gene report confirmed the targeted interaction between miR-326 and HDAC3 (*P* < 0.01, Figure 5(d)), which supported the above conjecture.

Western blot assay showed that overexpressing miR-326 significantly reduced the expression of HDAC3 and NF-*κ*B p65 and DNA-binding activity of NF-*κ*B p65 in chondrocytes, but significantly increased the levels of acetyl-STAT1, STAT1, and acetyl-NF-*κ*B p65 (*P* < 0.01, Figures 5(f) and 5(i)). However, overexpression of HDAC3 significantly reduced the levels of acetyl-STAT1, STAT1, and acetyl-NF-*κ*B p65, elevated by miR-326, and significantly increased the NF-*κ*B p65 expression level and DNA-binding activity decreased by miR-326 (*P* < 0.01, Figures 5(f) and 5(i)). These results suggest that miR-326 could promote the activation of STAT1/NF-*κ*B p65 signaling pathway by targeting HDAC3, thereby inhibiting pyroptosis of chondrocytes.

**Figure 6:**
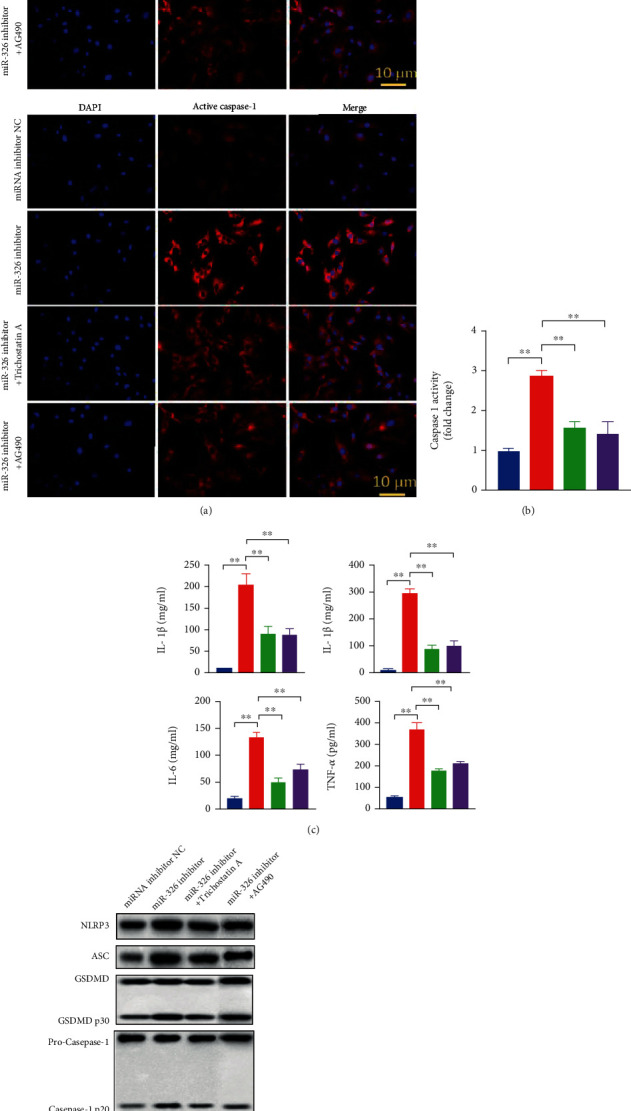
Inhibition of HDAC3 or STAT1/NF-*κ*B p65 signaling pathway counteracts pyroptosis induced by silence of miR-326 in chondrocytes. (a) Immunofluorescence results showed that inhibition of HDAC3 or STAT1/NF-*κ*B p65 could counteract the increase of GSDMD and cleaved Caspase-1 induced by miR-326 inhibitor in chondrocytes; (b) inhibition of HDAC3 or STAT1/NF-*κ*B p65 could counteract the increase of Caspase-1 activity induced by miR-326 inhibitor in chondrocytes; (c) inhibition of HDAC3 or STAT1/NF-*κ*B p65 could counteract the increase of proinflammatory factors (IL-1*β*, IL-18, IL-6, and TNF-*α*) induced by miR-326 inhibitor in chondrocytes; (d) Western blot results showed that inhibition of HDAC3 or STAT1/NF-*κ*B p65 could counteract the increase level of pyroptosis-related proteins (NLRP3, ASC, GSDMD, Caspase-1, IL-1*β*, and IL-18) induced by miR-326 inhibitor in chondrocytes; ^∗^*P* < 0.01, ^∗∗^*P* < 0.01.

We also treated chondrocytes with miR-326 inhibitors and inhibitors of HDAC3 or STAT1 signaling pathways, confirming the targeted regulation of miR-326 on HDAC3 and STAT1/NF-*κ*B p65 from another perspective. As shown in Figure 6, compared with the control, transfection with miR-326 inhibitor significantly increased the expression levels of pyroptosis-related proteins such as GSDMD, NLRP3, and ASC, as well as the activity and expression level of Caspase-1, and the levels of inflammatory factors IL-1*β*, IL-18, IL-6, and TNF-*α* (*P* < 0.01). Compared with the transfection of miR-326 inhibitor, the addition of HDAC3 or STAT1 inhibitor effectively alleviated the pyroptosis induced by miR-326 inhibitor, that is, decreased the protein expression levels of GSDMD, NLRP3, and ASC; Caspase-1 activity and expression; and levels of inflammatory factors IL-1*β*, IL-18, IL-6, and TNF-*α* (*P* < 0.01). These results confirmed that miR-326 regulates pyroptosis by targeting HDAC3 and STAT1 in chondrocytes.

### 3.4. In Vivo Experiments Confirmed That BMSC Exosomal Delivery of miR-326 Targeting HDAC3 and STAT1/NF-*κ*B p65 Inhibited OA Cartilage Pyroptosis

**Figure 7:**
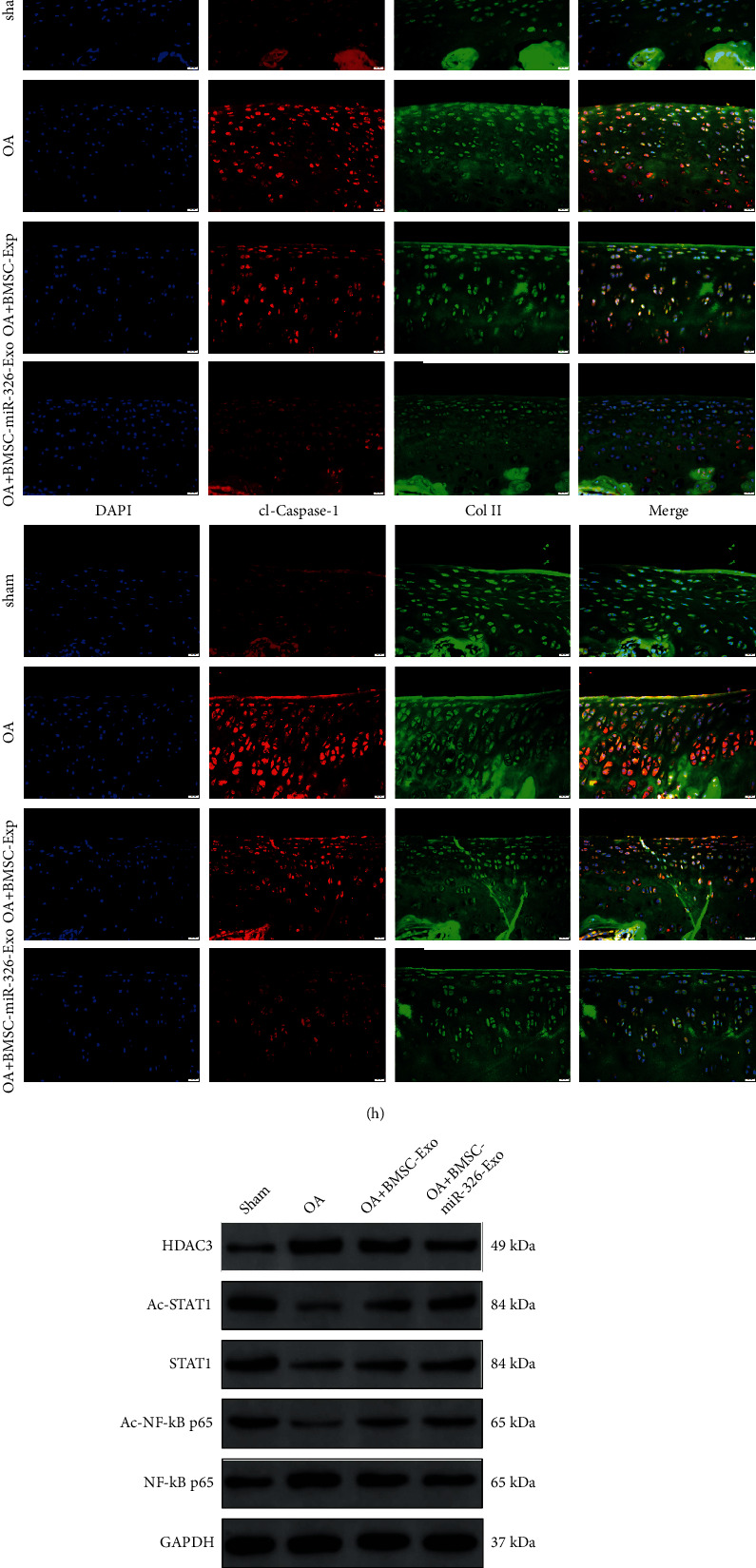
BMSCs exosomal deliver miR-326 to cartilage and inhibit cartilage pyroptosis via activating STAT1 by targeting HDAC3. (a) BMSC-Exos incubation reversed the reduction of ICRS scores induced by OA; (b) HE staining results of cartilage tissue in each group, bar = 20 *μ*m; (c): Safranin-O/Fast Green staining results of cartilage tissue in each group, bar = 20 *μ*m; (d) expression levels of miR-326, HDAC3, and chondrogenic-specific genes (COL2A1, SOX9, Agg, and Prg4); mRNAs in OA chondrocytes of each group and BMSC-Exos incubation reversed the down-regulated mRNA level of chondrogenic-specific genes in cartilage induced by OA; BMSC-Exos with overexpression of miR-326 had a more significant effect; (e) BMSC-Exos incubation counteracted the increase levels of proinflammatory factors (IL-1*β*, IL-18, IL-6, and TNF-*α*) in cartilage induced by OA, and BMSC-Exos with overexpression of miR-326 had a more significant effect; (f) BMSC-Exos incubation counteracted the increase of DNA-binding activity of NF-*κ*B p65 in cartilage induced by OA, and BMSC-Exos with overexpression of miR-326 had a more significant effect; (g) BMSC-Exos incubation counteracted the increase of Caspase-1 activity in cartilage induced by OA, and BMSC-Exos with overexpression of miR-326 had a more significant effect; (h) Immunofluorescence results showed that BMSC-Exos incubation counteracted the increase levels of GSDMD and cleaved Caspase-1 in cartilage induced by OA, and BMSC-Exos with overexpression of miR-326 had a more significant effect, bar = 20 *μ*m; (i) Western blot results showed that BMSC-Exos incubation counteracted the repression of STAT1/NF-*κ*B p65 signaling pathway in cartilage induced by OA, and BMSC-Exos with overexpression of miR-326 had a more significant effect; (j) Western blot results showed that BMSC-Exos incubation counteracted the increase levels of pyroptosis-related proteins (NLRP3, ASC, GSDMD, Caspase-1, IL-1*β*, and IL-18) in cartilage induced by OA, and BMSC-Exos with overexpression of miR-326 had a more significant effect; ^∗^*P* < 0.01, ^∗∗^*P* < 0.01.

To verify the mechanism of miR-326 in BMSC-Exos affecting OA cartilage pyroptosis at the animal level, we constructed a rat OA model and treated the rat model with exosomes extracted from normal BMSCs and BMSCs overexpressing miR-326, respectively. After 6 weeks of modeling, the cartilage sections of the knee joint of rats were taken for ICRS score, HE staining, and Safranin-O/fast green staining (Figures 7(a)–7(c)). The results showed that the ICRS score of articular cartilage of OA rats was significantly reduced (*P* < 0.01), and the surface of cartilage was rough. Absence of layers, reduced cell numbers, unclear and interrupted tide lines, and increased clusters of cells were observed in the OA articular cartilage. The staining with Safranin-O/Fast Green showed uneven, light, or dis-stained cartilage matrix in the OA articular cartilage. BMSC-Exos-treated cartilage showed significant ICRS score increase, cartilage layer thickness, increased cell number and orderly arrangement, relatively clear tide line, and deepened Safranin-O staining. It indicates the existence of abundant cartilage matrix and suggests cartilage tissue regeneration. The BMSC-Exos with overexpression of miR-326-treated OA articular cartilage showed better tissue regeneration.

The results of qRT-PCR showed that the expression level of HDAC3 in OA cartilage tissue was significantly increased, and the expression level of miR-326 and chondrogenic specific genes (COL2A1, SOX9, Agg, and Prg4) were significantly decreased (*P* < 0.01, Figure 7(d)). The expression levels of miR-326 and chondrogenic specific genes were significantly increased, and the level of HDAC3 was significantly decreased in BMSC-Exos-treated OA cartilage tissue, and the effect of BMSC-Exos overexpressing miR-326 was more significant (*P* < 0.05 or *P* < 0.01, Figure 7(d)). BMSC-Exos treatment significantly reduced the levels of GSDMD, cl-Caspase-1, Caspase-1 activity, proinflammatory cytokines, and pyroptosis-related protein expression in cartilage tissues significantly increased by OA (*P* < 0.01, Figures 7(e), 7(g), 7(h), and 7(j)). BMSC-Exos with overexpression of miR-326 also had a more significant effect (*P* < 0.01, Figures 7(e), 7(g), 7(h), and 7(j)).

Mechanically, OA significantly increased the levels of NF-*κ*B p65 expression and DNA-binding activity and significantly decreased the levels of acetyl-STAT1, STAT1, and acetyl-NF-*κ*B p65 (*P* < 0.01, Figures 7(f) and 7(i)). BMSCs-Exos treatment alleviated the effect of OA on cartilage tissue, and BMSCs-Exos overexpressing miR-326 had a more significant effect (*P* < 0.01, Figures 7(f) and 7(i)). These results confirm that delivery of miR-326 from BMSC-Exos targeting HDAC3 and STAT1/NF-*κ*B p65 ameliorates the pathogenesis of OA cartilage in vivo.

## 4. Discussion

OA is the most frequent form of arthritis, characterized by progressive degeneration in articular cartilage and chronic joint pain [37]. Owing to the limited understanding of the pathogenesis of OA, an urgent improvement of prevention and therapy is required in clinical practice. As pluripotent stem cells, BMSCs have played important roles in repairing and mitigating tissue damage in a variety of tissue damage models [38–40].

In recent years, increasing evidence has suggested that stem cells and precursor cells may play important roles in promoting tissue regeneration by activating surrounding cells through paracrine mechanisms [41, 42]. Most cells produce exosomes and play crucial roles in intercellular communication [43]. Moreover, exosomes have biological functions similar to those of the cells from which they were derived, and direct use of these nanoparticles has no obvious adverse effects, such as immune rejection or tumorigenicity [44]. In addition, exosomes can also promote the proliferation of chondrocytes in vitro [45]. Therefore, this study selected BMSC-Exos as the object to explore its regulatory effect on OA chondrocytes and the potential mechanism.

Our study found that incubation with BMSC-Exos can reverse the reduction of chondrocyte proliferation and migration caused by OA, as well as the decrease of chondrocyte-specific gene expression levels. The promoting effect of BMSC-Exos on the proliferation and migration of chondrocytes has also been supported in other studies [22, 23]. SOX9 is an important early transcription factor for chondrogenesis and chondrocyte differentiation, and it is a sensitive factor in articular cartilage tissue engineering, which plays an important role in chondrogenesis [46, 47]. Collagen type II (COL2A1), as a downstream target protein of Sox9, is an important component of the cartilage matrix and is considered the basis of structural strength to outside pressure [48]. Aggrecanases (Agg) belonging to the “A Disintegrin And Metalloproteinase with ThromboSpondin motifs” (ADAMTS) family of proteinases play a significant role in aggrecan depletion in osteoarthritic cartilage [49]. Aggrecanase-mediated aggrecan degradation is a significant event in early-stage OA. Proteoglycan 4 (Prg4), also known as Lubricin, is a surface-active mucus glycoprotein secreted by synovial joints, which acts as a lubricant in joints [50]. Maintenance of Prg4 expression prevents OA progression, which indicates that Prg4 plays an important role in maintaining the integrity of articular cartilage [51]. Our results showed that BMSC-Exos incubation significantly reversed the reduction of COL2A1, SOX9, Agg, and Prg4 expression levels induced by OA, which suggested that BMSC-Exos can protect chondrocyte homeostasis from osteoarthritis by promoting chondrogenesis and chondrocyte differentiation, cartilage matrix construction, cartilage degeneration, and synovial integrity. In vivo animal experiments have confirmed the role of BMSC-Exos in protecting cartilage integrity, maintaining the number of chondrocytes and cartilage matrix composition, and promoting cartilage tissue regeneration. Our results demonstrate the therapeutic effect of BMSC-Exos on OA, but its clinical application needs to be further studied.

Pyroptosis, a type of regulated cell death, occurs when pattern recognition receptors (PRRs) induce the activation of cysteine-aspartic protease 1 (caspase-1) or caspase-11, which can trigger the release of the pyrogenic cytokines interleukin-1*β* (IL-1*β*) and IL-18 [26]. Inflammasome is a polymeric protein complex and plays an important role in the pathogenesis of inflammation as a platform for caspase activation. Caspases are cysteine proteases that initiate or execute cellular programs and can induce inflammation or cell death, depending on its function [52]. Caspase-1 is the most fully characterized and is closely regulated by inflammasomes to process cytokines [53]. Activated caspase-1 is required for proteolysis and the release of cytokines IL-1*β* and IL-18 [54]. When damage is caused by endogenous stress or microbial infection, caspases release hazard-associated molecular patterns (DAMPs) or pathogen-associated molecular patterns (PAMPs), which trigger pattern recognition receptors (PRRs) [55]. Nucleotide-binding domain and leucine-rich repeat-containing (NLR) protein are one of the intracellular PRRs [56]. Activated caspase-1 triggers a typical inflammasome pathway leading to cell pyroptosis. Assembly of the NLRP3 inflammasome in response to PAMPs or DAMPs can lead to caspase-1-dependent release of proinflammatory cytokines and gasdermin D- (GSDMD-) mediated pyroptotic cell death. NLRP3 inflammasome activation requires upregulation of inflammasome components including NLRP3, Caspase-1, and pro-IL-1*β* by increasing the transcriptional activity of NF-*κ*B [57, 58]. Next, NLRP3 is activated by multiple upstream stimuli induced by a large number of PAMPs or DAMPs and then forms a complex with ASC and caspase-1. The complex cleaves the GSDMD and forms the GSDMD-N pores on the membrane to induce pyrolysis. We confirmed the inhibitory effect of BMSC-Exos on pyroptosis in OA by detecting changes in the expression levels of pyroptosis-related proteins including GSDMD, Caspase-1, NLRP3, ASC, IL-1*β*, and IL-18 in chondrocytes and cartilage, which is similar to the action pathway of other drugs [27, 28, 59].

In terms of mechanism, we found that BMSC-Exos treatment increased the expression level of miR-326 in chondrocytes, and the level of miR-326 directly affected the pyroptosis of chondrocytes. In this study, we used PHK26 to label exosomes and changed the level of miR-326 in exosomes by RNA interference techniques, confirming the role of miR-326 in exosomes entering chondrocytes. However, whether exosomes trigger endogenous secretion of miR-326 in chondrocytes remains to be further explored. MiR-326 has been proved to be involved in the regulation of pyroptosis in Parkinson's disease [35]. Histone deacetylase-3 (HDAC3) is ubiquitously expressed and conserved in a wide range of species [60]. HDAC3 can regulate various signaling pathways, including NF-*κ*B activity, and affect cell apoptosis [61]. It has been reported that miR-326 and HDAC3 can form a feedback loop to regulate the invasion and tumorigenic and angiogenic response to anticancer drugs [62]. Our experiment confirmed the targeted interaction between miR-326 and HDAC3, and miR-326 promoted the pyroptosis of chondrocytes by inhibiting HDAC3 and activating STAT1/NF-*κ*B p65 pathway. IL-4-STAT6 has polarization-specific epigenomic characteristics, and HDAC3-dependent macrophage response to inflammatory stimuli is reduced; inflammatory corpus activation and pyroptosis are reduced [63]. The role of HDAC3-dependent STAT1 and NF-*κ*B p65 pathways in the regulation of inflammatory response has also been demonstrated [64]. Valproic acid treatment inhibited HDAC3 expression and activity, enhanced STAT1, and acetylated NF-*κ*B p65 after spinal cord injury (SCI) [64]. The acetylation status of NF-*κ*B p65 and the NF-*κ*B p65 and STAT1 complex inhibit the transcriptional activity of NF-*κ*B p65 and attenuate the microglia-mediated central inflammatory response after SCI. Pyroptosis involves the release of intracellular proinflammatory factors (including IL-18 and IL-1*β*), and pyroptosis-regulated cell death plays a key role in the pathogenesis of various neurological diseases including SCI [65]. Transcription factor activity of NF-*κ*B is a key factor in activating NLRP3 inflammasome and pyroptosis. A melatonin and adipose study determined that nuclear factor *κ*B (NF-*κ*B) signaling is essential in GSDMD-mediated pyrophosis of adipose cells and that melatonin alleviates inflammatory body-induced adipose tissue spontaneous combustion in mice by blocking NF-*κ*B/GSDMD signaling [66]. Besides, NEK7 interacts with NLRP3 to modulate NLRP3 inflammasome activation, therefore, modulating the pyroptosis in mice [67]. Polyphyllin VI induced caspase-1-mediated pyroptosis via the induction of the ROS/NF-*κ*B/NLRP3/GSDMD signal axis in non-small-cell lung cancer [68]. Retinoic acid-inducible gene I (RIG-I) agonist therapy activates proinflammatory transcription factors STAT1 and NF-*κ*B and triggers external apoptotic pathways and pyroptosis, a highly immunogenic form of cell death in breast cancer cells [69]. Studies on murine Norovirus (MNV) suggest that protective STAT1 signaling controls viral replication and pathogenesis and that NLRP3 inflammasome activation and subsequent GSDMD-driven pyrodeath are contributors to MNV-induced immunopathology in susceptible STAT1-deficient mice [70]. A large number of studies all above have confirmed the key role of HDAC3 and STAT1/NF-*κ*B p65 in pyroptosis, and our results provide evidence that miR-326 plays a direct role in chondrocytes, and HDAC3 and STAT1/NF-*κ*B p65 affect pyroptosis. On the whole, miR-326 targeting HDAC3 activates the STAT1/NF-*κ*B p65 signaling pathway, inhibits the occurrence of pyroptosis, and protects chondrocytes from osteoarthritis.

## 5. Conclusion

BMSC-Exos can deliver miR-326 to chondrocytes and cartilage and ameliorate OA by targeting HDAC3 and STAT1//NF-*κ*B p65 to inhibit pyroptosis of chondrocytes and cartilage. Our findings provide a new mechanism instruction for BMSC-Exos in OA treatment.

## 6. Limitations

There are still some limitations to this study. First, due to the limited conditions of experimental equipment, we failed to directly observe and compare the morphological changes of chondrocyte pyroptosis and confirm the role of BMSC-Exos on pyroptosis. In addition, the therapeutic effect of BMSC-Exos remains to be validated in a clinical trial. All these limitations should be addressed in the future study.

## Data Availability

The original contributions presented in the study are included in the paper and supplementary material; further inquiries can be directed to the corresponding author.

## Supplementary Materials

Supplementary Materials Supplementary Table 1 is the primer sequences used for quantitative RT-PCR, and Supplementary Table 2 is the macroscopic evaluation criteria of (International Society for Chondroprosthesis) ICRS for cartilage repair.

## References

[1] Daugaard R., Tjur M., Sliepen M., Lipperts M., Grimm B., Mechlenburg I. (2018). Are patients with knee osteoarthritis and patients with knee joint replacement as physically active as healthy persons?. *Journal of Orthopaedic Translation*.

[2] Murray C. J. (2013). The state of US health, 1990-2010. *JAMA*.

[3] Hootman J. M., Helmick C. G. (2006). Projections of US prevalence of arthritis and associated activity limitations. *Arthritis and Rheumatism*.

[4] Liu-Bryan R., Terkeltaub R. (2015). Emerging regulators of the inflammatory process in osteoarthritis. *Nature Reviews Rheumatology*.

[5] van Meurs J. B., Uitterlinden A. G. (2012). Osteoarthritis year 2012 in review: genetics and genomics. *Osteoarthritis and Cartilage*.

[6] Gao B., Gao W., Wu Z. (2018). Melatonin rescued interleukin 1*β*-impaired chondrogenesis of human mesenchymal stem cells. *Stem Cell Research & Therapy*.

[7] Wang Y., Xu J., Zhang X. (2017). TNF- _*α*_ -induced LRG1 promotes angiogenesis and mesenchymal stem cell migration in the subchondral bone during osteoarthritis. *Cell Death & Disease*.

[8] Zhen G., Wen C., Jia X. (2013). Inhibition of TGF-*β* signaling in mesenchymal stem cells of subchondral bone attenuates osteoarthritis. *Nature Medicine*.

[9] Bornes T. D., Adesida A. B., Jomha N. M. (2014). Mesenchymal stem cells in the treatment of traumatic articular cartilage defects: a comprehensive review. *Arthritis Research & Therapy*.

[10] Zemljic M., Pejkovic B., Krajnc I., Kocbek L. (2015). Modern stem cell therapy: approach to disease. *Wiener Klinische Wochenschrift*.

[11] Colombo M., Raposo G., Thery C. (2014). Biogenesis, secretion, and intercellular interactions of exosomes and other extracellular vesicles. *Annual Review of Cell and Developmental Biology*.

[12] Raposo G., Stoorvogel W. (2013). Extracellular vesicles: exosomes, microvesicles, and friends. *Journal of Cell Biology*.

[13] Fang S. B., Zhang H. Y., Wang C. (2020). Small extracellular vesicles derived from human mesenchymal stromal cells prevent group 2 innate lymphoid cell-dominant allergic airway inflammation through delivery of miR-146a-5p. *Journal of Extracellular Vesicles*.

[14] Liu Y., Lin L., Zou R., Wen C., Wang Z., Lin F. (2018). MSC-derived exosomes promote proliferation and inhibit apoptosis of chondrocytes via lncRNA-KLF3-AS1/miR-206/GIT1 axis in osteoarthritis. *Cell Cycle*.

[15] Liu Y., Zou R., Wang Z., Wen C., Zhang F., Lin F. (2018). Exosomal KLF3-AS1 from hMSCs promoted cartilage repair and chondrocyte proliferation in osteoarthritis. *The Biochemical Journal*.

[16] Mao G., Zhang Z., Hu S. (2018). Exosomes derived from miR-92a-3p-overexpressing human mesenchymal stem cells enhance chondrogenesis and suppress cartilage degradation via targeting WNT5A. *Stem Cell Research & Therapy*.

[17] Wang Y., Yu D., Liu Z. (2017). Exosomes from embryonic mesenchymal stem cells alleviate osteoarthritis through balancing synthesis and degradation of cartilage extracellular matrix. *Stem Cell Research & Therapy*.

[18] Cosenza S., Ruiz M., Toupet K., Jorgensen C., Noël D. (2017). Mesenchymal stem cells derived exosomes and microparticles protect cartilage and bone from degradation in osteoarthritis. *Scientific Reports*.

[19] Kim Y. G., Choi J., Kim K. (2020). Mesenchymal stem cell-derived exosomes for effective cartilage tissue repair and treatment of osteoarthritis. *Biotechnology Journal*.

[20] Mianehsaz E., Mirzaei H. R., Mahjoubin-Tehran M. (2019). Mesenchymal stem cell-derived exosomes: a new therapeutic approach to osteoarthritis?. *Stem Cell Research & Therapy*.

[21] Tan S. S. H., Tjio C. K. E., Wong J. R. Y. (2021). Mesenchymal stem cell exosomes for cartilage regeneration: a systematic review of PreclinicalIn VivoStudies. *Tissue Engineering Part B: Reviews*.

[22] He L., He T., Xing J. (2020). Bone marrow mesenchymal stem cell-derived exosomes protect cartilage damage and relieve knee osteoarthritis pain in a rat model of osteoarthritis. *Stem Cell Research & Therapy*.

[23] Zhou X., Liang H., Hu X. (2020). BMSC-derived exosomes from congenital polydactyly tissue alleviate osteoarthritis by promoting chondrocyte proliferation. *Cell Death Discovery*.

[24] Boise L. H., Collins C. M. (2001). _Salmonella_ -induced cell death: apoptosis, necrosis or programmed cell death?. *Trends in Microbiology*.

[25] Fink S. L., Cookson B. T. (2005). Apoptosis, pyroptosis, and necrosis: mechanistic description of dead and dying eukaryotic cells. *Infection and Immunity*.

[26] An S., Hu H., Li Y., Hu Y. (2020). Pyroptosis plays a role in osteoarthritis. *Aging and Disease*.

[27] Zu Y., Mu Y., Li Q., Zhang S. T., Yan H. J. (2019). Icariin alleviates osteoarthritis by inhibiting NLRP3-mediated pyroptosis. *Journal of Orthopaedic Surgery and Research*.

[28] Hu J., Zhou J., Wu J. (2020). Loganin ameliorates cartilage degeneration and osteoarthritis development in an osteoarthritis mouse model through inhibition of NF-*κ*B activity and pyroptosis in chondrocytes. *Journal of Ethnopharmacology*.

[29] Huang Y., Tan F., Zhuo Y. (2020). Hypoxia-preconditioned olfactory mucosa mesenchymal stem cells abolish cerebral ischemia/reperfusion-induced pyroptosis and apoptotic death of microglial cells by activating HIF-1*α*. *Aging*.

[30] Wang J., Ren H., Yuan X., Ma H., Shi X., Ding Y. (2018). Interleukin-10 secreted by mesenchymal stem cells attenuates acute liver failure through inhibiting pyroptosis. *Hepatology Research*.

[31] Liang C., Liu Y., Xu H. (2021). Exosomes of human umbilical cord MSCs protect against hypoxia/reoxygenation-induced pyroptosis of cardiomyocytes via the miRNA-100-5p/FOXO3/NLRP3 pathway. *Frontiers in Bioengineering and Biotechnology*.

[32] Wang G., Yuan J., Cai X. (2020). HucMSC-exosomes carrying miR-326 inhibit neddylation to relieve inflammatory bowel disease in mice. *Clinical and Translational Medicine*.

[33] Azimi M., Ghabaee M., Moghadasi A. N., Izad M. (2019). Altered expression of miR-326 in T cell-derived exosomes of patients with relapsing-remitting multiple sclerosis. *Iranian Journal of Allergy, Asthma, and Immunology*.

[34] Bai Z. Z., Li H. Y., Li C. H., Sheng C. L., Zhao X. N. (2020). M1 macrophage-derived exosomal MicroRNA-326 suppresses hepatocellular carcinoma cell progression via mediating NF-*κ*B signaling pathway. *Nanoscale Research Letters*.

[35] Zhang Q., Huang X. M., Liao J. X. (2021). LncRNA HOTAIR promotes neuronal damage through facilitating NLRP3 mediated-pyroptosis activation in Parkinson’s disease via regulation of miR-326/ELAVL1 axis. *Cellular and Molecular Neurobiology*.

[36] Kumar P., Gogulamudi V. R., Periasamy R., Raghavaraju G., Subramanian U., Pandey K. N. (2017). Inhibition of HDAC enhances STAT acetylation, blocks NF-*κ*B, and suppresses the renal inflammation and fibrosis inNpr1haplotype male mice. *American Journal of Physiology. Renal Physiology*.

[37] Glyn-Jones S., Palmer A. J. R., Agricola R. (2015). Osteoarthritis. *The Lancet*.

[38] Islam M. N., Das S. R., Emin M. T. (2012). Mitochondrial transfer from bone-marrow-derived stromal cells to pulmonary alveoli protects against acute lung injury. *Nature Medicine*.

[39] Ding J., Wang X., Chen B., Zhang J., Xu J. (2019). Exosomes derived from human bone marrow mesenchymal stem cells stimulated by deferoxamine accelerate cutaneous wound healing by promoting angiogenesis. *BioMed Research International*.

[40] Zhang Q., Li Q., Zhu J. (2019). Comparison of therapeutic effects of different mesenchymal stem cells on rheumatoid arthritis in mice. *PeerJ*.

[41] Kim J. Y., Song S. H., Kim K. L. (2010). Human cord blood-derived endothelial progenitor cells and their conditioned media exhibit therapeutic equivalence for diabetic wound healing. *Cell Transplantation*.

[42] Zhang M., Malik A. B., Rehman J. (2014). Endothelial progenitor cells and vascular repair. *Current Opinion in Hematology*.

[43] Théry C., Ostrowski M., Segura E. (2009). Membrane vesicles as conveyors of immune responses. *Nature Reviews Immunology*.

[44] Burger D., Viñas J. L., Akbari S. (2015). Human endothelial colony-forming cells protect against acute kidney injury: role of exosomes. *The American Journal of Pathology*.

[45] Zhu Y., Wang Y., Zhao B. (2017). Comparison of exosomes secreted by induced pluripotent stem cell-derived mesenchymal stem cells and synovial membrane-derived mesenchymal stem cells for the treatment of osteoarthritis. *Stem Cell Research & Therapy*.

[46] Zhang H., Zhao X., Zhang Z., Chen W., Zhang X. (2013). An immunohistochemistry study of Sox9, Runx2, and Osterix expression in the mandibular cartilages of newborn mouse. *BioMed Research International*.

[47] Soltz M. A., Basalo I. M., Ateshian G. A. (2003). Hydrostatic pressurization and depletion of trapped lubricant pool during creep contact of a rippled indenter against a biphasic articular cartilage layer. *Journal of Biomechanical Engineering*.

[48] Zwickl H., Niculescu-Morzsa E., Halbwirth F. (2016). Correlation analysis of SOX9, -5, and -6 as well as COL2A1 and aggrecan gene expression of collagen I implant-derived and osteoarthritic chondrocytes. *Cartilage*.

[49] Huang K., Wu L. D. (2008). Aggrecanase and aggrecan degradation in osteoarthritis: a review. *The Journal of International Medical Research*.

[50] Coles J. M., Zhang L., Blum J. J. (2010). Loss of cartilage structure, stiffness, and frictional properties in mice lacking PRG4. *Arthritis and Rheumatism*.

[51] Chavez R. D., Sohn P., Serra R. (2019). Prg4 prevents osteoarthritis induced by dominant-negative interference of TGF-ß signaling in mice. *PLoS One*.

[52] Schroder K., Tschopp J. (2010). The inflammasomes. *Cell*.

[53] Martinon F., Burns K., Tschopp J. (2002). The Inflammasome: A Molecular Platform Triggering Activation of Inflammatory Caspases and Processing of proIL-*β*. *Molecular Cell*.

[54] Broz P., von Moltke J., Jones J. W., Vance R. E., Monack D. M. (2010). Differential requirement for caspase-1 autoproteolysis in pathogen-induced cell death and cytokine processing. *Cell Host & Microbe*.

[55] Lamkanfi M., Dixit V. M. (2014). Mechanisms and functions of inflammasomes. *Cell*.

[56] Takeuchi O., Akira S. (2010). Pattern recognition receptors and inflammation. *Cell*.

[57] Xing Y., Yao X., Li H. (2017). Cutting edge: TRAF6 mediates TLR/IL-1R signaling-induced nontranscriptional priming of the NLRP3 inflammasome. *Journal of Immunology*.

[58] Bauernfeind F. G., Horvath G., Stutz A. (2009). Cutting edge: NF-*κ*B activating pattern recognition and cytokine receptors license NLRP3 inflammasome activation by regulating NLRP3 expression. *Journal of Immunology*.

[59] Yu H., Yao S., Zhou C. (2021). Morroniside attenuates apoptosis and pyroptosis of chondrocytes and ameliorates osteoarthritic development by inhibiting NF-*κ*B signaling. *Journal of Ethnopharmacology*.

[60] Mahlknecht U., Emiliani S., Najfeld V., Young S., Verdin E. (1999). Genomic organization and chromosomal localization of the human histone deacetylase 3 gene. *Genomics*.

[61] Chen L. (2001). Duration of nuclear NF-kappa B action regulated by reversible acetylation. *Science*.

[62] Kim Y., Kim H., Park H. (2014). miR-326-Histone Deacetylase-3 Feedback Loop Regulates the Invasion and Tumorigenic and Angiogenic Response to Anti-cancer Drugs. *The Journal of Biological Chemistry*.

[63] Czimmerer Z., Daniel B., Horvath A. (2018). The transcription factor STAT6 mediates direct repression of inflammatory enhancers and limits activation of alternatively polarized macrophages. *Immunity*.

[64] Chen S., Ye J., Chen X. (2018). Valproic acid attenuates traumatic spinal cord injury-induced inflammation via STAT1 and NF-*κ*B pathway dependent of HDAC3. *Journal of Neuroinflammation*.

[65] al Mamun A., Wu Y., Monalisa I. (2021). Role of pyroptosis in spinal cord injury and its therapeutic implications. *Journal of Advanced Research*.

[66] Liu Z., Gan L., Xu Y. (2017). Melatonin alleviates inflammasome-induced pyroptosis through inhibiting NF-*κ*B/GSDMD signal in mice adipose tissue. *Journal of Pineal Research*.

[67] Chen X., Liu G., Yuan Y., Wu G., Wang S., Yuan L. (2019). NEK7 interacts with NLRP3 to modulate the pyroptosis in inflammatory bowel disease via NF-*κ*B signaling. *Cell Death & Disease*.

[68] Teng J. F., Mei Q. B., Zhou X. G. (2020). Polyphyllin VI induces caspase-1-mediated pyroptosis via the induction of ROS/NF-*κ*B/NLRP3/GSDMD signal axis in non-small cell lung cancer. *Cancers*.

[69] Elion D. L., Jacobson M. E., Hicks D. J. (2018). Therapeutically active RIG-I agonist induces immunogenic tumor cell killing in breast cancers. *Cancer Research*.

[70] Dubois H., Sorgeloos F., Sarvestani S. T. (2019). Nlrp3 inflammasome activation and Gasdermin D-driven pyroptosis are immunopathogenic upon gastrointestinal norovirus infection. *PLoS Pathogens*.

