# Supplementary material for: BMSC-Derived Exosomes Ameliorate Osteoarthritis by Inhibiting Pyroptosis of Cartilage via Delivering miR-326 Targeting HDAC3 and STAT1//NF-κB p65 to Chondrocytes
Source: Mediators Inflamm. 2021 Nov 2;2021:9972805. doi: 10.1155/2021/9972805 (PMC8577926; doi:10.1155/2021/9972805)
Supplement: Supplementary Materials — Supplementary Table 1 is the primer sequences used for quantitative RT-PCR, and Supplementary Table 2 is the macroscopic evaluation criteria of (International Society for Chondroprosthesis) ICRS for cartilage repair. [file 9972805.f1.zip › 9972805.f1.docx]

**Supplementary Table 1** Primer sequences used for quantitative RT-PCR

| Gene | Sequences (5’-3’) |
| --- | --- |
| miR-326 | F: ACTGTCCTTCCCTCTGGGC  R: AATGGTTGTTCTCCACTCTCTCTC |
| HDAC3 | F: ACCAATATGCAAGGCTTCACCAA  R: GCCTGTGTAACGCGAGCAGA |
| COL2A1 | F: GCCCAACTGGCAAACAAGGAGAC  R: GCAGGGCCAGAAGTACCC TGATC |
| SOX9 | F: GGAGATGAAATCTGTTCTGGGAATG  R: TTGAAGGTTAACTGCTGGTGTTCTG |
| Agg | F: GATGTTCCCTGCAATTACCACCTC  R: TGATCTCATACCGGTCCTTCTTCTG |
| Prg4 | F: GAA CGT GCT ATA GGA CCT TC  R: CAG ACT TTG GAT AAG GTC TGC C |
| GAPDH | F: CCTGGAGAAACCTGCCAAGTAT  R: TAGCCCAGGATGCCCTTTAGT |
| U6 | F: CTCGCTTCGGCAGCACATATACT  R: ACGCTTCACGAATTTGCGTGTC |

F, forward; R, reverse.
