# Supplementary material for: BMSC-Derived Exosomes Ameliorate Osteoarthritis by Inhibiting Pyroptosis of Cartilage via Delivering miR-326 Targeting HDAC3 and STAT1//NF-κB p65 to Chondrocytes
Source: Mediators Inflamm. 2021 Nov 2;2021:9972805. doi: 10.1155/2021/9972805 (PMC8577926; doi:10.1155/2021/9972805)
Supplement: Supplementary Materials — Supplementary Table 1 is the primer sequences used for quantitative RT-PCR, and Supplementary Table 2 is the macroscopic evaluation criteria of (International Society for Chondroprosthesis) ICRS for cartilage repair. [file 9972805.f1.zip › 9972805.f2.docx]

**Supplementary Table 2** ICRS macroscopic evaluation of cartilage repair

| Cartilage repair assessment | Standard | Points |
| --- | --- | --- |
| Degree of defect repair | In level with surrounding cartilage  75% repair of defect depth  50% repair of defect depth  25% repair of defect depth  0% repair of defect depth | 4  3  2  1  0 |
| Integration to border zone | Complete integration with surrounding cartilage  Demarcating border < 1 mm  3/4 of graft integrated, 1/4 with a notable border > 1 mm  1/2 of graft integrated, 1/2 with a notable border > 1 mm  From no contact to 1/4 of graft integrated with surrounding cartilage | 4  3  2  1  0 |
| Macroscopic appearance | Intact smooth surface  Fibrillated surface  Small, scattered fissures or cracs  Several, small or few but large fissures  Total degeneration of grafted area | 4  3  2  1  0 |
| Overall repair assessment | Grade I: normal 12  Grade II: nearly normal 11 ~ 8  Grade III: abnormal 7 ~ 4  Grade IV: severely abnormal 3 ~ 1 | 12  11~8  7~4  3~1 |
